# Impact on Pregnancies in South Brazil from the Influenza A (H1N1) Pandemic: Cohort Study

**DOI:** 10.1371/journal.pone.0088624

**Published:** 2014-02-18

**Authors:** André Anjos da Silva, Tani Maria Schilling Ranieri, Fernanda Duarte Torres, Fernanda Sales Luiz Vianna, Graziella Rangel Paniz, Paula Baptista Sanseverino, Paulo Dornelles Picon, Pietro Baptista de Azevedo, Marta Haas Costa, Lavinia Schuler-Faccini, Maria Teresa Vieira Sanseverino

**Affiliations:** 1 Teratogen Information Service, Medical Genetics Service, Hospital de Clinicas de Porto Alegre, Porto Alegre, Brazil; 2 Post-Graduate Program in Genetics and Molecular Biology at the Federal University of Rio Grande do Sul, Brazil; 3 Epidemiological Surveillance, Department of Health, Rio Grande do Sul, Brazil; 4 Department of Internal Medicine, Hospital de Clínicas de Porto Alegre (HCPA), Porto Alegre, Rio Grande do Sul, Brazil; Alberta Provincial Laboratory for Public Health/University of Alberta, Canada

## Abstract

**Introduction:**

The emergence of a new subtype of the influenza virus in 2009 generated interest in the international medical community, the media, and the general population. Pregnant women are considered to be a group at risk of serious complications related to the H1N1 influenza virus. The aim of this study was to evaluate the outcomes and teratogenic effects of pregnancies exposed to the H1N1 virus during the Influenza A epidemic that occurred in the state of Rio Grande do Sul in 2009.

**Methods:**

This is an uncontrolled prospective cohort study of pregnant women with suspected symptoms of Influenza A who were reported in the Information System for Notifiable Diseases – Influenza (SINAN-Influenza) during the epidemic of 2009 (database from the state of Rio Grande do Sul, Brazil). There were 589 cases of pregnant women with suspected infection. Among these, 243 were tested by PCR and included in the analysis. The main outcome measures were: maternal deaths, pregnancy outcome, stillbirths, premature births, low birth weight, congenital malformations, and odds ratios for H1N1+ and non-H1N1 pregnant women.

**Results:**

There were one hundred and sixty-three (67%) confirmed cases of H1N1, 34 cases (14%) of seasonal Influenza A and 46 (19%) who were negative for Influenza A. There was no difference between the three groups in clinical parameters of the disease. There were 24 maternal deaths — 18 of them had H1N1. There were 8 stillbirths — 5 were children of H1N1 infected mothers. There were no differences in perinatal outcomes.

**Conclusions:**

The present data do not indicate an increase in teratogenic risk from exposure to the influenza A (H1N1) virus. These results will help to strengthen the data and clarify the health issues that arose after the pandemic.

## Introduction

The emergence of a new subtype of the influenza virus at the end of March 2009 generated interest in the international medical community, the media, and the general population. The influenza A (H1N1) epidemic began in Mexico and rapidly spread to other countries around the world [1] and was declared a pandemic by the World Health Organization (WHO) approximately two months after the first cases appeared [2]. In Brazil, it was declared pandemic in mid-July 2009.

The Influenza A H1N1 virus is the product of multiple genetic rearrangements among strains of influenza that had previously been circulating. Some of these were unique to swine and birds and became capable of infecting humans [3].

Pregnant women are considered to be a group at risk of serious complications related to the H1N1 influenza virus, with high morbidity and mortality observed in both previous [4]–[6] and recent [7], [8] epidemics. Studies of the consequences for pregnancy and the fetus, both for the influenza and its treatment, are also important in the context of public health.

The only known specific treatment, which is supported by recent evidence, is the use of the neuraminidase inhibitors zanamivir and oseltamivir, of which only the latter is available in Brazil.

Rio Grande do Sul is the southernmost state of Brazil. It occupies 3% of the national territory and has a population of 10,914,128 inhabitants (2009), which corresponds to about 6% of the national population. The climate in this state has specific characteristics that are different from the rest of the country. For example, it has the lowest temperatures for the Brazilian winter (humid subtropical climate with average temperatures between 15°C and 18°C) [9].

During the Influenza A (H1N1) pandemic in 2009 there were 44,544 cases confirmed in Brazil and 2,051 deaths recorded. In Rio Grande do Sul, there were 8,314 reported cases, 3,576 confirmed cases (8% of total cases in Brazil), and 297 deaths (14.5% of national deaths) [10], [11].

As for effects on the embryo/fetus, there are still few studies on the teratogenic potential of the influenza virus. It has been suggested that there is a potential increase in the risk of defects in neural tube closure associated with maternal hyperthermia [12]. Few studies focus on the teratogenic effects of this virus on the embryo/fetus [13]–[17].

Analyses of the effects of Influenza A H1N1 on pregnancies during the pandemic of 2009 have focused primarily on maternal mortality and morbidity, in part due to the immediacy of the pandemic and the speed with which the series of cases were published [15].The follow-up of women after hospital admission, with a focus on pregnancy outcomes, has now been further investigated [15], [16], [18]. Some of these studies confirm the worse outcomes in pregnant women infected with H1N1 Influenza virus and proved the benefits of vaccination and treatment for this condition [18], [19].

In South America, only one Brazilian study, which was conducted with 57 hospitalized women, has shown the clinical characteristics and pregnancy outcomes of pregnant women infected with Influenza A (H1N1) during the 2009 pandemic [20].

The aim of this study was to evaluate the outcomes,including teratogenic effects, of pregnancies exposed to the H1N1 virus during the Influenza A epidemic that occurred in the state of Rio Grande do Sul in 2009.

## Materials and Methods

This is an uncontrolled prospective cohort study that evaluated pregnancies with exposure to the 2009 H1N1 Influenza virus. The sample consisted of pregnant women with suspected symptoms of Influenza A who were reported in the Information System for Notifiable Diseases - Influenza (SINAN-Influenza) during the epidemic of 2009 (database from the state of Rio Grande do Sul).

SINAN is maintained by the reporting and investigation of cases of diseases and conditions that appear on the national list of diseases for which notification is compulsory. Besides the Individual Notification Form (FIN), the system also provides an Individual Investigation Form (FII), which is a research guideline that enables the identification of the source of infection and the mechanisms of disease transmission. Most of the information about the patient and his clinical symptoms are collected at the time of notification [21].

Confirmation of the infection and detection of the viral subtype was done (when possible) through a biological sample (nasopharyngeal swab) in order to perform the real time polymerase chain reaction (RT-PCR), in accordance with the RT-PCR protocol for the detection and characterization of Swine Influenza from the Centers for Disease Control and Prevention (CDC) [22].

Pregnancy follow-up was conducted by a trained team via telephone, between July 2009 and June 1, 2011. If three phone calls were unsuccessful and a visit to a patient's home could not be arranged, a follow-up was considered to be lost. Data from medical records or the Live Birth Certificate (LBC) would then be used to verify the pregnancy outcome data. The LBC is the standard document of the Ministry of Health and its use is mandatory in Brazil. It includes data to be filled in about the mother (age, gravidity), the pregnancy (gestational age), type of delivery, and the newborn child (birth weight, Apgar score, congenital malformations, etc.). The LBC does not include information about pregnancy complications or comorbidities like pregnancy-related hypertension/preeclampsia, gestational diabetes, etc.).

A home visit, when possible, was performed by a team member who used a questionnaire identical to that used in the phone interviews. This follow-up by personal contact received direct support from the State Center for Health Surveillance (CEVS).

### Participants

During the Influenza A H1N1 epidemic in 2009, there were 589 cases of pregnant women suspected of infection that were reported to the SINAN-Influenza database for Rio Grande do Sul.

Patients were excluded from this study if they did not have a confirmed pregnancy, if it was not possible to contact them after notification, or if they did not have a PCR performed.

For data analysis, we included only patients with infection confirmed by PCR laboratory analysis. Follow-ups were done either by phone, home visit, assessment of medical records, or via the LBC information.

### Case Ascertainment

An influenza-like illness (ILI) was defined as the presence of at least one of the common flu symptoms (rhinorrhea, conjunctivitis, sore throat, cough, shortness of breath, headache, backache, muscle ache, joint pain, fever, nausea, vomiting, diarrhea, or asthenia), in accordance with the widely used definition, and this was reported to the SINAN-Influenza database.

**Figure 1:**
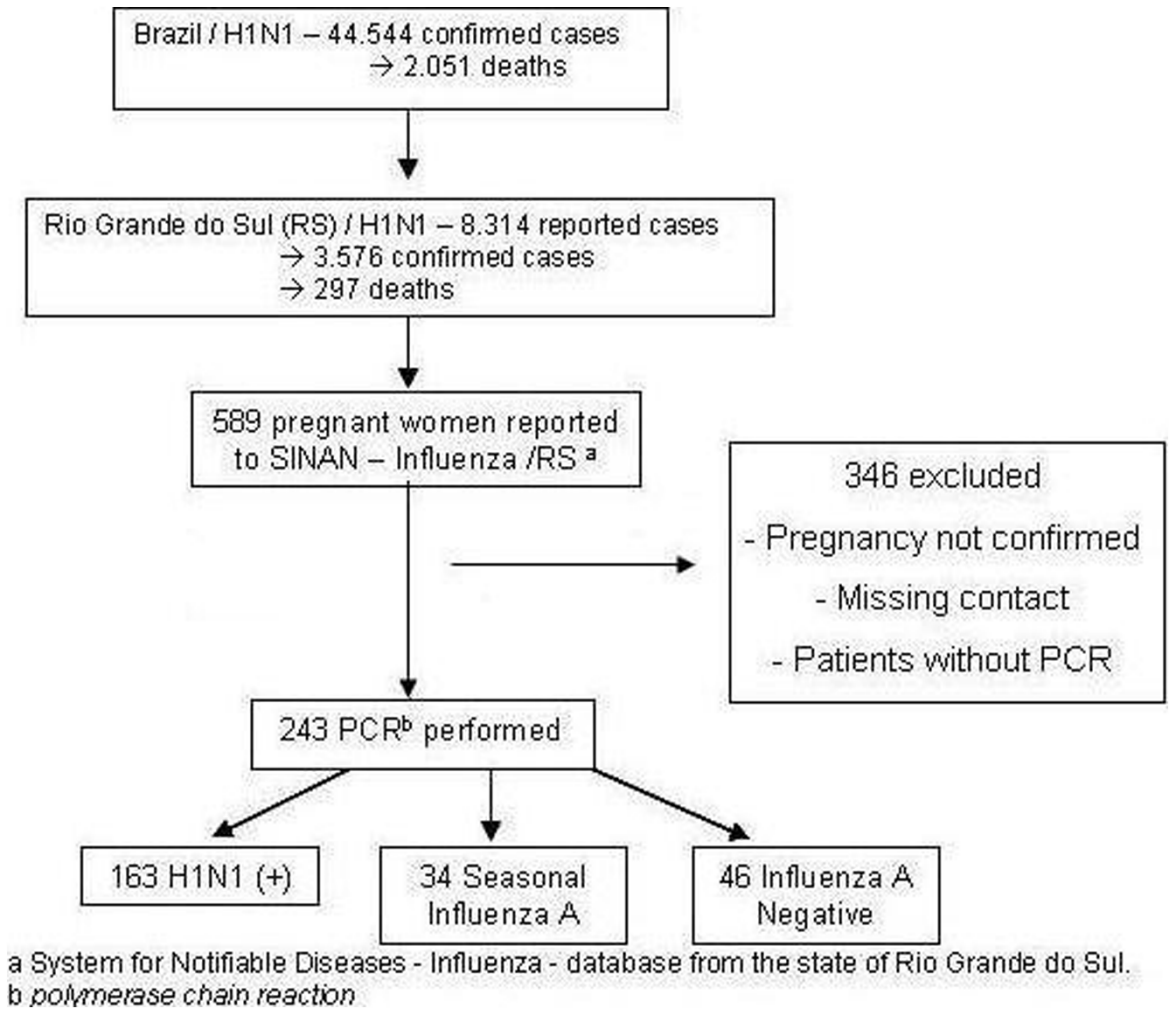
H1N1 cases reported, confirmed, and with a PCR performed, in Rio Grande do Sul. Footnote: a. Information System for Notifiable Diseases-Influenza (SINAN-Influenza): database for the state of Rio Grande do Sul. b. Polymerase chain reaction.

A confirmed case was defined as an ILI with a positive Influenza A (H1N1) PCR, and is denoted as H1N1+. Non-H1N1 Influenza cases were those that were seasonal Influenza A or that were negative for Influenza A (see Figure 1). Considering that the PCR techniques were specific for H1N1 and seasonal Influenza A, it was not possible to identify other agents among the Influenza A negative subgroup.

### Statistical Methods

We evaluated clinical parameters related to the pregnancy (gestational age in the notification, pharmaceutical form of the oseltamivir phosphate used, other medications taken, outcome of pregnancy: miscarriage or birth, and type of birth) and the newborn child (prematurity, low weight, presence of congenital malformations, and neonatal complications). A descriptive analysis was done of the samples, with means and standard deviations for both the quantitative variables of the clinical parameters and the frequency of the categorical variables. The analyses were done using Fisher's exact test followed by the Z-test with Bonferroni correction, One-way ANOVA, or the Kruskal-Wallis test, as appropriate. The level of significance was set at 5% for all variables and the program used for the analyses was SPSS for Windows, version 18.0.

### Ethics Statement

The research was approved by the Research Ethics Committee of the Research and Post-Graduate Group at the Hospital de Clinicas de Porto Alegre (CEP/GPPG-HCPA).

At the beginning of the telephone interview, consent was requested for confirmation of the information available in the records of SINAN-Influenza or for collection of new data, if necessary. Informed consent was conducted verbally, since most of the interviews were conducted via telephone. The patient answered “Yes, I authorize” or “No” to the question “Do you consent to talk by phone with a medical team and provide some data about your flu syndrome episode and your pregnancy?”. The same verbal informed consent was applied to cases of personal interviews. All procedures were approved by GPPG and the clinical investigation was conducted according to the principles expressed in the Declaration of Helsinki.

## Results

During the Influenza A H1N1 epidemic in 2009, there were 589 cases of pregnant women suspected of infection who reported to the SINAN-Influenza database for Rio Grande do Sul.

From the follow-ups done, 243 PCR test results were obtained. There were one hundred and sixty three (67%) confirmed cases of Influenza A H1N1, 34 cases (14%) of seasonal Influenza A, and 46 (19%) negative for Influenza A (Figure 1). Contact was made directly in about 40% of cases. The remaining follow-ups were facilitated by medical records or Live Birth Certificates.

The accurate gestational age at the time of infection was obtained in 83 follow-ups. Three women (1.2%) were in the first trimester (0–11 weeks), 27 (11%) were in the second trimester (12–23 weeks) and 53 (21.8%) patients were in the third trimester (≥24 weeks).

Use of oseltamivir phosphate was identified in 137 patients (56.3%). Adverse reactions such as vomiting, nausea, abdominal pain, diarrhea, drowsiness, confusion, dizziness, headache, and irritability were reported. There were no serious adverse events in this study population. There was no difference when comparing the three groups in terms of adverse reactions presented. In the analysis of symptoms reported after use of the medication, in comparison with the H1N1+ subgroup only nausea was more frequent in patients without H1N1 confirmed by PCR (OR: 0.36, 95% CI: 0.15–0.86, p<0.01).

Hospitalization was required for 107 of the patients. In the H1N1+ subgroup, 70 (42.9%) patients required hospitalization. The other 37 admissions consisted of 6 (17.6%) in the seasonal Influenza A subgroup and 31 (67.4%) in the Influenza A negative subgroup. The proportion of hospitalizations is significantly different for each group; in other words, there was a predominance of hospitalizations in the Influenza A negative subgroup, followed by the H1N1+ subgroup, and finally the seasonal Influenza A subgroup (p<0.05, see Table 1). Of these 107 patients, 58 (54.2%) were in the third trimester of pregnancy (38 H1N1+).

**Table 1:** Clinical parameters presented by pregnant women with influenza-like illness in south Brazil.

	H1N1+ (n = 163)		Seasonal Influenza A (n = 34)		Influenza A negative (n = 46)	
	n	%	n	%	n	%
**Hospitalization** a	70	42.9*	6	17.6*	31	67.4*
**Mechanical ventilation** a	27	16.6	4	11.7	10	21.7
**Antibiotic use** a	50	30.7	7	20.5	21	45.6
**Maternal deaths** a	18	11	2	5.8	2	4.3

a Fisher's exact test followed by Z-test with Bonferroni correction.

*Significance level was set at p<0.05.

The clinical parameters are presented in Table 1 and show no differences between the subgroups.

Twenty-four maternal deaths (5.6%) were recorded, 18 of these (75%) were H1N1+ patients. Two deaths were in the seasonal Influenza A subgroup and 2 deaths in the Influenza A negative subgroup (Table 1). The remaining two deaths were recorded for pregnant women for whom a PCR was not done. Only two of these 24 patients were not admitted to the intensive care unit and did not require mechanical ventilation (both were from the H1N1+ subgroup). Fifteen of these women (13 were H1N1+) had received oseltamivir and there was an average of 3.8 days between the onset of symptoms and treatment. Case-fatalities did not differ between the subgroups. The total number of deaths from Influenza (ICD-10 J09 [Influenza due to certain identified influenza viruses], J10 [Influenza due to other identified influenza virus], and J11 [Influenza due to unidentified influenza virus] for women of childbearing age (15–49 years) in 2009 in the state of Rio Grande do Sul was 117. Of these, 97 were attributed to ICD-10 J09 (Influenza due to a certain identified influenza virus). In 2008 and 2010 there were no deaths in this subgroup of women that could be attributed to the ICDs mentioned above. In 2011, there were 4 deaths in this age group (2 due to ICD-10 J09) [21]. These ICDs were used for comparison purposes of the maternal deaths between the years with and without H1N1 pandemic.

Regarding pregnancy outcomes, we obtained 236 live births. Eight stillbirths were reported — five of these were for H1N1+ pregnant women, one was from the seasonal Influenza A subgroup, and the other two were for pregnant women for whom research about the virus was not done. There were three miscarriages — one occurred in the Influenza A negative subgroup and two in pregnant women without PCR, so there was no difference between the subgroups (Table 2). Information regarding the type of delivery was obtained in 232 follow-ups. There was no difference between the subgroups when comparing vaginal birth and elective cesarean sections. However, differences were observed regarding the need for an emergency cesarean section (Table 2).

**Table 2:** Pregnancy outcomes in H1N1 confirmed cases and non-H1N1 influenza cases in south Brazil.

	H1N1+ (n = 163)		Seasonal Influenza A (n = 34)		Influenza A negative (n = 46)	
	N	%	n	%	n	%
**Live births** a	158	97.0	33	97.0	45	97.8
**Stillbirths** a	5	3.0	1	3.0	0	0
**Miscarriages** a	0	0	0	0	1	2.2
***Delivery method***						
**Vaginal births** a	50	30.7	11	32.3	10	21.7
**Elective cesarean sections** a	67	41.1	14	41.1	11	23.9
**Emergency cesarean sections** a	39	23.9*	9	26.4	21	45.6*
***Gestational age at birth***						
**Mean age in weeks (±SD)** b	37.0 (±4.5)	-	37.2 (±4.2)	-	36.8 (±3.8)	-
**<37 weeks** a	38	23.3	9	26.4	15	32.6
***APGAR score (mean)***						
**1^st^ minute** c	7.6	-	8.2	-	8.3	-
**5^th^ minute** c	8.8	-	9.1	-	8.9	-
**Birth weight in grams (mean±SD)** b	3128 (±643)	-	2893 (±493)	-	2998 (±598)	-

a Fisher's exact test followed by Z-test with Bonferroni correction.

b One-way ANOVA.

c Kruskal-Wallis test.

*Significance level was set at p<0.05 for all tests.

Gestational age at birth was obtained in 222 follow-ups. The mean gestational age did not differ between the subgroups. The weight of the newborns was, on average, 3128 grams (±643) for those born to H1N1+ mothers, 2893 grams (±493) for the seasonal Influenza A subgroup, and 2998 grams (±598) for the Influenza A negative subgroup.

There were no reports of major complications or malformations for the newborns.

## Discussion

By observing the previous pandemics and epidemics, as well as seasonal influenza, pregnant women have been identified as a group at risk of higher morbidity and mortality. During the epidemic of 1957–58, 10% of deaths from influenza in New York (USA) occurred in pregnant women. In Minnesota (USA), 50% of deaths among women of reproductive age occurred during pregnancy [5], [6].

In the influenza A (H1N1) pandemic of 2009, pregnant women were among the first cases and first deaths reported, representing 13% of deaths reported to the centers for control and prevention of diseases in the USA [7]. The first studies of this pandemic revealed a hospitalization rate among pregnant women that was approximately four times higher than for the general population [7]. Therefore, pregnancy is considered a risk factor for severe complications related to infection with Influenza A (H1N1). Despite this, there are few studies focusing on the teratogenic effects of this infection.

In this study, the gestational and maternal outcomes were almost the same when correlated with the Influenza A H1N1 virus. These results differ from other studies which show poor pregnancy outcomes, such as increased perinatal mortality and prematurity [15], and low birth weight [16], in the presence of infection by the H1N1 virus during maternity. The study of Pierce et al. [15] suggests that, in comparison with uninfected pregnancies, there is an increased risk of poor pregnancy outcomes for women infected with 2009/H1N1. Different from those previous studies showing worse outcomes on H1N1 infected pregnant women compared to healthy pregnant controls, our investigation compared the H1N1 infection with pregnant women with ILI, and we did not find differences in outcomes among the groups. This apparently contradictory results might be explained by the use of different control groups. Another study has also suggested equal severe effects resulting from H1N1 infection both in pregnant compared to non-pregnant women [17].

The present study may have been unable to detect poor outcomes due to the sample sizes and the severity of maternal disease in the three subgroups used for comparison. It should be noted that in the H1N1 exposed group in our sample the prevalence of deaths (18/163, 11%), although statistically non-significant, was almost the double of the non-H1N1 influenza groups (2/34, 5.8% and 2/46, 4.3%) (Table 2). In the same line of thought, we cannot discard possible differential effects on maternal mortality related to H1N1, since in 2009, the year of the H1N1 pandemic, there were 117 deaths attributed to influenza in women of childbearing age (15–49 years) in the state of Rio Grande do Sul, compared to none in the years 2008 and 2010. This in not a direct evidence, but should be considered that potentially much more deaths had occurred due to H1N1.

Additional information was obtained about the safety of oseltamivir, given that there had been no report of any major complication or abnormality among the outcomes for pregnant women who had used this drug. These data are very important due to the large number of outcomes that could be assessed in this study and they reinforce safety data already published in other papers [23], [24].

It is important to emphasize that the follow-ups of 424 pregnant women were done from a sample of 589 pregnant women reported to SINAN-Influenza in 2009. This represents a follow-up rate of 72%, an average that is much higher than for many other studies published on this subject, which have among their limitations a small number of follow-ups (Table 3) [15], [25]–[29]. Of these follow-ups, we included in the analysis 243 patients who performed PCR in order to confirm or rule out infection.

**Table 3:** Previous studies of pregnancy outcomes among women infected with the 2009 H1N1.

Study	No. of pregnant women reported	No. and (%) actually infected'	Pregnancy outcome	No. affected	Maternal deaths	Antiviral treatment
**LOUIE, 2010 (USA) ** [**26**]	94	32 (34)	Miscarriage	02	06	81%
**ANZIC, 2010 (AUS/NZ) ** [**29**]	64	61 (95)	Miscarriage	02	06	81%
			Stillbirth	04		
			Premature delivery	22		
			Low birth weight	18		
**HEWAGAMA, 2010 (AUS) ** [**28**]	43	15 (35)	Premature delivery	06	01	77%
		24 (56)	Stillbirth/neonatal death	03		
**SISTON, 2010 (USA) ** [**25**]	788	169 (21)	Premature delivery	51	30	84%
		200 (25)	Miscarriage	08		
**CREANGA, 2010 (USA) ** [**27**]	62	40 (65)	Premature delivery	06	02	50%
			Neonatal death	02		
**DUBAR, 2010 (FRA) ** [**14**]	314	146 (46)	Stillbirth	02	03	95%
			Loss of pregnancy (<24weeks)	04		
			Premature delivery	26		
			Low birth weight	22		
**GÉRARDIN, 2010 (FRA) ** [**13**]	271	139 (51)	Premature delivery	17	-	86%
			Congenital anomalies	01		
**PIERCE, 2011 (UK) ** [**15**]	272	256 (94)	Stillbirth	07	-	-
			Loss of pregnancy (<24weeks)	05		
			Premature delivery	58		
			Congenital anomalies	08		
**PRESENT STUDY, 2012 (BRA)**	589	243 (41)	Stillbirth	08	24	56%
			Loss of pregnancy (<24weeks)	03		
			Congenital anomalies	01		

One limitation of our study is that we did not collect some clinical information about the respiratory symptoms exhibited by the pregnant women, such as the presence of hyperthermia (and maximum temperature) and viral load, which could account for some of the adverse outcomes reported. However, this limitation is also present in other recent studies on this subject [15].

The difficulty in performing follow-ups is due to the number of mobile phones used as the main contact — phone numbers change and phones are lost. Moreover, at the time of the Influenza A (H1N1) pandemic, the consultations were mostly conducted in places adapted for high patient demand, thus it was more difficult and there was a greater margin of error at the time of completing the personal data of the pregnant woman being treated.

Previous experience with pregnant women exposed to Influenza A suggests that the maternal outcomes are worse in this subgroup of women. This justified the use of antiviral treatment and vaccinations against the Influenza A H1N1 virus for these patients. Regardless, our results were not sufficient to support this; therefore, it is important to emphasize that more studies are needed, especially ones that correlate maternal exposure to the H1N1 virus with neonatal outcomes, so that we can further clarify the health issues that arose after this pandemic.
